# Predicting amyloid proteins using attention-based long short-term memory

**DOI:** 10.7717/peerj-cs.2660

**Published:** 2025-02-07

**Authors:** Zhuowen Li

**Affiliations:** Punan Branch of Renji Hospital, Shanghai Jiao Tong University, Shanghai, China

**Keywords:** Amyloid, Transformers, Deep learning, LSTM, Alzheimer, Attention

## Abstract

Alzheimer’s disease (AD) is one of the genetically inherited neurodegenerative disorders that mostly occur when people get old. It can be recognized by severe memory impairment in the late stage, affecting cognitive function and general daily living. Reliable evidence confirms that the enhanced symptoms of AD are linked to the accumulation of amyloid proteins. The dense population of amyloid proteins forms insoluble fibrillar structures, causing significant pathological impacts in various tissues. Understanding amyloid protein’s mechanisms and identifying them at an early stage plays an essential role in treating AD as well as prevalent amyloid-related diseases. Recently, although several machine learning methods proposed for amyloid protein identification have shown promising results, most of them have not yet fully exploited the sequence information of the amyloid proteins. In this study, we develop a computational model for *in silico* identification of amyloid proteins using bidirectional long short-term memory in combination with an attention mechanism. In the testing phase, our findings showed that the model developed by our proposed method outperformed those developed by state-of-the-art methods with an area under the receiver operating characteristic curve of 0.9126.

## Introduction

In many organs and tissues, amyloid proteins tend to flock to create insoluble aggregates of different sizes. These amyloid aggregates can continuously build up to produce intracellular protein inclusions or extracellular plaques, most notably as a component of disease processes. They are mostly made up of $\beta$-sheet structures and have a fibrillary shape when aggregated (Rambaran & Serpell, 2008). Disease-related amyloid proteins, found as plaques inside the central nervous system, include those involved in the pathological trigger or growth of Alzheimer’s disease (AD). Amyloid-beta (A $\beta$) plaques, neurofibrillary tangles, and brain shrinkage are the hallmarks of AD, a neurodegenerative condition. It causes dementia most often (Beach et al., 2012; Matsui et al., 2008) and has a major societal effect (Nichols et al., 2019; Matsui et al., 2008). However, since AD’s clinical symptoms might overlap with those of other illnesses, including frontotemporal lobar degeneration or late-onset mental disorders, making a clinical diagnosis of the disease can be difficult (Wang et al., 2025). These illnesses may occasionally coexist with AD and have comparable clinical signs and symptoms (Beach et al., 2012; Hampel et al., 2021; Wang et al., 2022). In experimental laboratories, histochemical dyes (*e.g*., Congo Red, Thioflavin T, *etc*.) are frequently used for pathological examination to investigate whether amyloid proteins are present in tissues. Besides, mass spectrometry can be used to identify amyoid proteins and classify their type of accumulation and patterns (Vrana et al., 2009). Since the structures and sizes of proteins building up amyloid fibrils are highly varied (López de la Paz et al., 2002), more advanced techniques are required to detect amyloid’s mark and its type in particular tissues. Among known modern assays, liquid chromatography-tandem mass spectrometry (LC-MS/MS) is considered the most effective, stable, and accurate one. However, as a high-level technique with expensive chemicals used, applying it requires large budgets, skilled experimenters, and a longer time. To accelerate the screening process and save budgets, computational biologists have developed various *in silico* models using current computer-aided advances. Several studies have utilized computational methods to predict the propensity for $\beta$-amyloid formation (Orlando et al., 2019), determine the aggregation-prone areas (APRs) of amyloid proteins (Maurer-Stroh et al., 2010; Thangakani et al., 2014), and identify amyloidogenicity (Palato et al., 2019). Aggregation Nucleation Prediction in Peptides and Proteins (ANuPP), by Prabakaran et al. (2021), was developed using ensemble learning. This tool was designed to flexibly identify putative APRs in peptides and proteins, overcoming the shortcomings of many existing models at the time. With the continuous growth of empirical data on pharmaceutical, chemical, and biological sequences (Mendez et al., 2018), coupled with advancements in artificial intelligence (AI), there has been a growing focus over the past several decades on developing more efficient models for amyloid proteins, particularly leveraging bio-molecular representations in bio-cheminformatics (Nguyen-Vo et al., 2024; Chen et al., 2024). Advanced AI techniques have been utilized to analyze neuroimaging data and patient histories (Chen et al., 2017), aiding in the diagnosis and treatment of various neurological diseases (Pham et al., 2019). Attention mechanisms have been leveraged to improve model interpretability by focusing on the most relevant features in imaging data (Chen et al., 2021; Wu et al., 2024). Furthermore, hybrid models integrating recurrent neural networks (RNN) with attention mechanisms have been developed to capture temporal dynamics and essential features in patient data, resulting in enhanced prediction accuracy for AD-related outcomes (Li & Ma, 2022).

## Related work

For years, many machine learning models have constructed to predict sequences using different representations, such as amino acid composition (AAC) (Bhasin & Raghava, 2004), composition transition distribution (CTDC) based on the percentage of particular amino acid property groups (Li et al., 2006; Dubchak et al., 1995; Chen et al., 2018), amphiphilic pseudo-amino acid composition (APAAC) (Chou, 2011), composition transition distribution difference (CTDD) based on distribution of amino acid properties in sequences (Chiti & Dobson, 2006; Vrana et al., 2009), and Dipeptide deviation from expected mean (DDE) (Saravanan & Gautham, 2015; Wang et al., 2020). RFAmyloid, developed by Niu et al. (2018), is one of the pioneered model designed to distinguish amyloid proteins from ordinary ones. It was created using random forest and different encoding schemes for sequence information extraction. They collected and curated protein sequence samples to obtain a dataset with 165 amyloid and 382 non-amyloid proteins for modeling. iAMY-SCM, by Charoenkwan et al. (2021), was developed the Scoring Card method to predict and analyze amyloid proteins. In their proposed method, a streamlined weighted-sum function was used in combination with thr propensity scores computed for dipeptides. iAMY-SCM was reported to achieve as good performance as RFAmyloid did based on metrics evaluated in cross-validation and independent testing (Charoenkwan et al., 2021). Charoenkwan et al. (2022) proposed AMYPred-FRL, an ensemble model with better performance reported. They combined six common machine learning algorithms, including logistic regression, *k*-nearest neighbors (k-NN), support vector machine (SVM), maximum gradient boosting (MGB), extremely randomized trees, and random forest (RF), and ten different sequence-based feature descriptors to construct 60 base models. In prediction, these base models returned 60 probabilistic features, which were then fed to the final meta-model (Charoenkwan et al., 2022). Most recently, Yang, Liu & Zhang (2023) proposed ECAmyloid, another ensemble machine learning model that detects amyloid proteins using diverse sequence-derived features. The sequence-based features combined information on the composition, evolution, and structure of the sequences. To select the most suitable models for ensemble learning, they used an enhanced classification selection method. The prediction outcomes of the meta-learner were based on the decision of all voters (base models) (Yang, Liu & Zhang, 2023).

## Materials and Methods

### Dataset

We used a refined dataset collected from Niu et al. (2018) for model development and evaluation, consisting of 165 amyloid proteins (positive samples) and 382 non-amyloid proteins (negative samples). The raw data was used without any transformations prior to the main analysis. The dataset was then randomly sampled to create two sets of data: a training set and an independent test set for modeling and performance assessment. The training set contains 132 positive samples and 305 negative samples, while the test set contains 33 positive samples and 77 negative samples. From the training set, we created a validation set to find the optimal point for our model. Table 1 provides information of sampled data in the training, validation, and test sets.

**Table 1:** Information on datasets used for model training, validation, and testing.

Dataset	Training	Validation	Test
Positive	117	15	33
Negative	276	29	77
Total	393	44	110

### Justification for model type used

Existing computational approaches for amyloid protein identification each have their own strengths and weaknesses, highlighting the need for more effective methods. In this study, we propose an attention-based bidirectional long short-term memory (Bi-LSTM) model, which offers significant advantages over traditional models. The Bi-LSTM architecture is specifically chosen for its ability to capture bidirectional dependencies in sequence data, crucial for understanding the structural information of proteins (Nguyen-Vo et al., 2022). Unlike traditional LSTMs, which process data in a single direction and capture only past context, Bi-LSTMs process sequences in both forward and backward directions, allowing the model to utilize preceding and succeeding information. By integrating an attention layer, the model can selectively focus on important features, enhancing its accuracy for amyloid protein prediction. This approach addresses limitations in existing methods by leveraging the detailed information contained within protein structures.

### Selection method

To develop and evaluate the proposed model, we utilized a refined dataset collected from Niu et al. (2018). The model’s architecture was carefully selected and optimized based on its validation performance on this dataset. We benchmarked the proposed model against other machine learning models trained on multiple commonly used feature sets, such as amino acid composition (AAC) (Wei et al., 2018), amino acid pair composition (APAAC) (Chen, Cheong & Siu, 2021), composition transition distribution (CTDC) (Zhao et al., 2022), composition transition distribution difference (CTDD) (Thi Phan et al., 2022), and dynamic positional encoding (DPE) (Li & Ma, 2022), to ensure a comprehensive assessment. AAC and APAAC provide foundational compositional insights, while CTDC and CTDD offer more detailed spatial information. DPE introduces a dynamic approach to positional encoding, making it suitable for advanced machine learning applications. Each method has its strengths and is selected based on the specific requirements of studies. Furthermore, we compared our approach with current computational frameworks for amyloid protein identification, including ECAmyloid (Yang, Liu & Zhang, 2023), AMYPred-FRL (Charoenkwan et al., 2022), iAMY-SCM (Charoenkwan et al., 2021), and RFAmyloid (Niu et al., 2018). This comparative analysis helped in selecting and validating the Bi-LSTM model with attention as the most effective architecture for our study.

### Assessment metrics

The area under the receiver operating characteristic curve (AUROC) was chosen as the primary metric for model assessment due to its ability to evaluate the overall performance of the model across all possible classification thresholds, making it a robust indicator of discriminative ability. Additionally, balanced accuracy (BA), Matthews correlation coefficient (MCC), and F1 score (F1) were computed at the default threshold of 0.5 to provide a comprehensive evaluation of the model’s performance. BA accounts for imbalances in the dataset by considering both sensitivity and specificity, MCC provides a balanced measure of model quality even with imbalanced classes, and F1 score offers insight into the trade-off between precision and recall. Together, these metrics offer a well-rounded assessment of the model’s performance under various aspects of classification accuracy.

### Other benchmarking models

To fairly assess the performance of our model, we compared it to a series of machine learning models developed using commonly used feature sets. We selected five learning algorithms, including *k*-NN (Fix & Hodges, 1989), Logistic Regression (LR) (Tolles & Meurer, 2016), SVM (Cortes & Vapnik, 1995), RF (Breiman, 2001), and eXtreme Gradient Boosting (XGB) (Chen & Guestrin, 2016), combined with five feature sets comprising AAC (Bhasin & Raghava, 2004), APAAC (Chou, 2011), CTDC (Li et al., 2006; Dubchak et al., 1995; Chen et al., 2018), CTDD (Chiti & Dobson, 2006; Vrana et al., 2009), and DPE (Saravanan & Gautham, 2015; Wang et al., 2020) to create 25 machine learning models. We trained these models using the same training set as used for training our model. The hyperparameters of these models were determined based on the validation set. Besides machine learning models, we also implemented several baseline deep learning models, including RNN and gated recurrent units (GRU), both with and without the integration of an attention (Att) mechanism.

To further assess the effectiveness of our model in practice, we compared it with other existing computational frameworks, including RFAmyloid (Niu et al., 2018), iAMY-SCM (Charoenkwan et al., 2021), AMYPred-FRL (Charoenkwan et al., 2022), and ECAmyloid (Yang, Liu & Zhang, 2023). For the RFAmyloid and iAMY-SCM frameworks, we accessed their web servers and uploaded our test samples to run the prediction tasks and then collected the results. Since these web servers predicted the classes of the samples (either amyloid protein or non-amyloid protein), we could only calculate balanced accuracy, MCC, and F1 score. For the AMYPred-FRL and ECAmyloid frameworks, we reimplemented them using the source codes provided by the authors and evaluated them on the same independent test set to report the results.

### Our proposed method

#### Bidirectional long short-term memory

Long short-term memory (LSTM), belonging to the family of RNNs, is designed to tackle the problem of gradient vanishing when the models are forced to learn long-distance sequential data (Hochreiter & Schmidhuber, 1997). To address this problem, gating mechanisms (forget, input, and output gates) and memory cells are employed to control flow of information. LSTM can maintain and update its cell state over long periods. LSTM’s design helps it effectively deal with many tasks from language processing to time series prediction. The mathematical expression of the *Forget* gate is as follows:

(1) $${f_t} = \delta ({W_{forget}}\left[ {{h_{t - 1}},{X_t}} \right] + {b_{forget}}),$$

where $\delta$ denote the sigmoid activation function, ${X_t}$ is the input vector at timestep $t$, and ${h_{t - 1}}$ is the hidden vector from the previous timestep $t - 1$. The weights ${W_f}$ and bias values ${b_f}$ are randomly initialized and gradually updated when the model learns. The mathematical expression of *Input* and *Output* gates are defined as:

(2) $${i_t} = \delta ({W_{input}}\left[ {{h_{t - 1}},{X_t}} \right] + {b_{input}}),$$

(3) $${O_t} = \delta ({W_{output}}\left[ {{h_{t - 1}},{X_t}] + {b_{output}}} \right).$$

The candidate memory cell ( ${L_t}$) contributes to adjusting the information in the current cell state ( ${C_t}$). The output ${O_t}$ and current cell state ( ${C_t}$) are used to create the current hidden stage ( ${h_t}$), as follows:

(4) $${L_t} = \tanh ({W_{cell}}[{h_{t - 1}},{X_t}] + {b_{cell}}),$$

(5) $${C_t} = {f_t} \times {C_{t - 1}} + {i_t} \times {L_t},$$

(6) $${h_t} = {O_t} \times \tanh \left( {{C_t}} \right).$$

#### Attention layer

Our attention layer accept hidden states $H = [{h_1},{h_2},{h_3}, \cdots ,{h_n}]$ returned from the LSTM layer. The vectors of hidden states ${h_k}$ are then nonlinearly transformed to create activated output $U = [{u_1},{u_2},{u_3}, \cdots ,{u_n}]$ using the Tanh function:

(7) $${u_k} = \tanh ({{\bf{W}}_k}{h_k} + {b_k}),$$

where ${{{W}}_k}$ denotes the weight matrices and ${b_k}$ refers to the offset quantity at timestep $k$. Certain operational factors during the shield tunneling process have a significant impact on the direction and location of the shield tunnel, thus they should be given additional consideration. The attention mechanism is applied to create the attention weight matrix ${\alpha _k} = [{\alpha _1},{\alpha _2},{\alpha _3},{\alpha _4} \cdots ,{\alpha _n}]$. This matrix stores important information of all single intermediate states, described as:

(8) $${\alpha _k} = {{\exp \left( {{{u}}_k^T{{{u}}_{\mathrm{s}}}} \right)} \over {\sum\nolimits_{k = 1}^n {\exp } ({{u}}_k^T{{{u}}_{\mathrm{s}}})}},$$

where ${\alpha _k}$ refers to the normalized attention weight at timestep $k$ and ${{{u}}_{\mathrm{s}}}$ is the time series attention matrix which are randomly initialized. Finally, for each input sample, a computed weighted sum of hidden states is obtained to create an attention vector *V* for the next stage:

(9) $$V = \sum\limits_k^n {{\alpha _k}} {h_k}.$$

#### Model architecture

**Figure 1:**
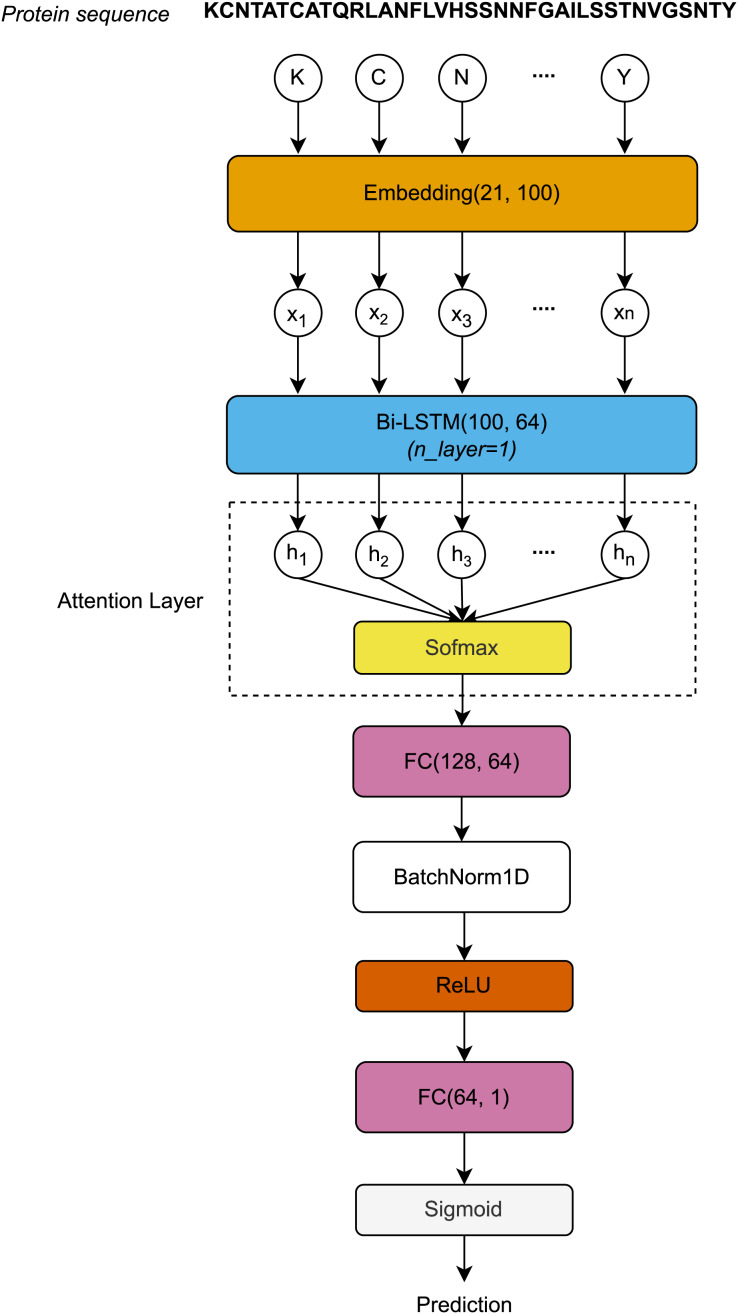
Architecture of the proposed model.

The protein sequences, each represented as a distinct chain of letters, are encoded by the Embedding layer to form a sequence vector $X = ({X_1},{X_2},{X_3},{X_4}, \ldots ,{X_t})$. First, the sequence vector length *L* is embedded after passing through the Embedding layer. The Embedding layer is defined by a vocabulary of 21 letters and an embedding dimension of 100. The embedding matrices, with dimensions $L \times 100$, are then input to the Bi-LSTM layer. After processing through the Bi-LSTM layer, the hidden dimension vectors at all timesteps are used to compute the attention scores using the Softmax function. The attention outputs, with dimensions $1 \times 128$, are then passed through the first Fully Connected (FC) layer, normalized using 1-Dimensional Batch Normalization (BatchNorm1D) (Ioffe & Szegedy, 2015), and activated by the rectified linear unit (ReLU) function. Finally, the activated outputs are transferred to the second FC layer, where they are activated by the Sigmoid function to produce the final prediction outcomes. Figure 1 visualizes the architecture of the proposed model.

### Experimental settings

All the experiments were performed using a computer running Windows 11 Home, with an Intel Core i7-13700F CPU (16 cores/24 threads, max turbo 5.2 GHz). The computer is equipped with 2 $\times$ 16 GB RAM for temporary memory. All deep learning models were trained using the PyTorch platform (version 2) with CUDA Toolkit version 11.7. All libraries are compatible with Python 3.11.

We trained our models with the training set and monitored the modeling phase to find the optimal epoch for the model based on the validation losses. The optimization process was controlled using the Adam optimizer (Kingma & Ba, 2014). A learning rate of 0.001 was chosen for the entire modeling phase. Binary cross-entropy was used as the loss function for the training process. The model was trained over 40 epochs, and the loss function was found to converge at epoch 23.

## Results and discussion

Table 2 summarizes the performance of all implemented models, including model developed using our methods and those developed with other machine learning ones.

**Table 2:** Performance of our model against other machine learning models.

Model	Features	AUROC	BA	MCC	F1
	AAC	0.7627	0.6385	0.4073	0.3386
	APAAC	0.8322	0.7403	0.4805	0.4805
$k$-NN	CTDC	0.7470	0.5887	0.252	0.2147
	CTDD	0.7227	0.6926	0.4884	0.4472
	DDE	0.8213	0.7186	0.4372	0.4372
	AAC	0.7052	0.5000	0	0
	APAAC	0.8304	0.7359	0.4577	0.4561
LR	CTDC	0.6348	0.5238	0.1340	0.0643
	CTDD	0.7879	0.7684	0.5416	0.5415
	DDE	0.8855	0.7965	0.5621	0.5547
	AAC	0.8386	0.7792	0.5946	0.5890
	APAAC	0.8351	0.6926	0.4103	0.4064
RF	CTDC	0.7737	0.6840	0.4024	0.3953
	CTDD	0.8996	0.8225	0.6256	0.6234
	DDE	0.8579	0.7229	0.4638	0.4619
	AAC	0.8520	0.7597	0.5345	0.5333
	APAAC	0.8170	0.7424	0.4735	0.4726
SVM	CTDC	0.7218	0.6299	0.3086	0.2927
	CTDD	0.7696	0.7013	0.4881	0.4581
	DDE	0.8467	0.7489	0.4898	0.4894
	AAC	0.8142	0.7684	0.5416	0.5415
	APAAC	0.8339	0.7165	0.4454	0.4444
XGB	CTDC	0.7859	0.6688	0.3747	0.3662
	CTDD	0.8863	0.8052	0.6104	0.6104
	DDE	0.8819	0.7597	0.5345	0.5333
	RNN	0.8930	0.8455	0.6236	0.6222
	RNN-Att	0.8914	0.8455	0.6290	0.6288
Deep learning	GRU	0.8874	0.8636	0.6682	0.6667
	GRU-Att	0.8937	0.8818	0.7212	0.7210
	Ours	0.9126	0.8788	0.7454	0.7447

It can be observed that XGB is a promising model that can effectively learn features from protein sequences, as its metric values are remarkably greater than those evaluated for $k$-NN and SVM models. The AUROC values of the XGB model trained with the DDE and CTDD feature sets are 0.8819 and 0.8863, respectively. Additionally, the RF model trained with the DDE feature set has higher predictive power than models trained with the other feature sets, with an AUROC value of 0.8855. Compared to these machine learning models, our model achieves the highest performance with an AUROC value of 0.9126. The balanced accuracy and F1 score of our model also outperform the other models, with values of 0.8788 and 0.7447, respectively.

The comparative analysis between our model and the other computational frameworks is shown in Table 3. The results indicate that the model developed using our proposed method has better performance compared to the existing frameworks, with an AUROC value of 0.9126 and a balanced accuracy of 0.8788. Regarding the MCC, our model remains the best-performing model with a value of 0.7454. Our model’s F1 score is also greater than that of other models, with a value of 0.7447.

**Table 3:** Performance of our model against other computational frameworks.

Model	AUROC	BA	MCC	F1
RFAmyloid	–	0.8364	0.6104	0.6104
iAMY-SCM	–	0.8182	0.5600	0.5595
AMYPred-FRL	0.9055	0.8636	0.6682	0.6667
ECAmyloid	0.8937	0.8455	0.6236	0.6222
Ours	0.9126	0.8788	0.7454	0.7447

## Model’s robustness examination

The performance of our model was evaluated over 10 random modeling trials, as summarized in Table 4. Across these trials, the model demonstrates robust performance, with an average AUROC value of 0.9167, indicating a high capability for distinguishing between classes. Similarly, the mean balanced accuracy of 0.8898 reflects a strong balance in sensitivity and specificity. The average MCC value of 0.7447 further confirms the reliability of the model in terms of binary classification correlation. Finally, the averaged F1 score of 0.7438 highlights its consistent precision and recall balance. These results demonstrate the stability and efficacy of the model, as evidenced by the low standard deviations across all metrics.

**Table 4:** Performance of our model over 10 random modeling trials.

Trial	AUROC	BA	MCC	F1
1	0.9126	0.8788	0.7454	0.7447
2	0.9104	0.8846	0.7386	0.7379
3	0.9297	0.8942	0.7469	0.7451
4	0.9094	0.8846	0.7300	0.7293
5	0.9356	0.9038	0.7667	0.7659
6	0.9036	0.8846	0.7251	0.7244
7	0.9320	0.8942	0.7515	0.7498
8	0.9097	0.8846	0.7345	0.7338
9	0.9163	0.9038	0.7781	0.7781
10	0.9072	0.8846	0.7300	0.7293
Mean	0.9167	0.8898	0.7447	0.7438
SD	0.0115	0.0087	0.0170	0.0171

## Limitations and future work

The proposed method, while demonstrating strong performance in amyloid protein identification using Bi-LSTM combined with an attention mechanism, does have certain drawbacks. One primary concern is the model’s reliance on sequence information alone. Although this approach captures essential patterns within protein sequences, it may overlook critical structural and functional information that could enhance predictive accuracy. For instance, complex folding processes often occur in amyloid proteins, and their 3D structures and interactions with other biomolecules closely influence their pathological effects. By focusing solely on sequence data, the model could miss these aspects, potentially limiting its generalizability to different types of amyloid proteins or other related neurodegenerative diseases.

Another limitation is the potential risk of overfitting, particularly given the complexity of the Bi-LSTM and attention mechanisms used. Overfitting occurs when a model learns to perform exceptionally well on the training data but fails to generalize to new, unseen data. If the training dataset is not sufficiently large or diverse, it exacerbates this risk. Moreover, the computational complexity of the model can pose practical challenges, especially when dealing with large-scale datasets or deploying it in real-time diagnostic settings. The interpretability of the model’s decisions is also a concern, as the intricate layers of Bi-LSTM and attention mechanisms can obscure the reasoning behind predictions, making it difficult for researchers or clinicians to trust and verify the model’s outputs.

For future work, it would be beneficial to explore the integration of additional types of data, such as structural information from 3D protein folding patterns or interaction data with other biomolecules. This multi-modal approach could provide a more comprehensive understanding of amyloid proteins and potentially improve the model’s accuracy and generalizability. Another promising direction is the development of techniques to enhance model interpretability, such as attention visualization or layer-wise relevance propagation, which would allow researchers to better understand which parts of the protein sequence contribute most to the predictions. Additionally, efforts should be made to reduce the model’s computational demands, possibly through model optimization or the use of more efficient architectures, to facilitate its deployment in practical, real-world scenarios. Finally, ongoing validation with larger and more diverse datasets will be crucial to ensuring the model’s robustness and applicability across different populations and conditions.

## Conclusion

In this work, we presented an effective model for the *in silico* screening of amyloid proteins using attention-based Bi-LSTM. The introduction of an attention layer helps the model selectively focus on essential information and extract important features from amyloid protein sequences. Experimental results indicated that the model developed using our proposed method improved performance on the independent test set compared to other machine learning models as well as existing computational frameworks. Other metrics, including balanced accuracy, MCC, and F1 score, also demonstrated the robustness of the proposed method. This method can be modified to suit various dataset sizes and applied to address similar problems.

## Supplemental Information

10.7717/peerj-cs.2660/supp-1 Supplemental Information 1 The Python code and data used in the experiments.

10.7717/peerj-cs.2660/supp-2 Supplemental Information 2 An introduction and explanation of the code, and steps for implementation.

10.7717/peerj-cs.2660/supp-3 Supplemental Information 3 Raw data results from all baseline machine learning models.

10.7717/peerj-cs.2660/supp-4 Supplemental Information 4 Raw data results from all comparing models.

## Additional Information and Declarations

### Data Availability

The following information was supplied regarding data availability:

The Python code and data used in the experiments are available in the Supplemental File.

The data from Niu et al. (2018) is available at GitHub: https://github.com/KOALA-L/ECAmyloid/tree/main/dataset.

## References

[ref-1] Beach TG, Monsell SE, Phillips LE, Kukull W (2012). Accuracy of the clinical diagnosis of Alzheimer disease at national institute on aging Alzheimer disease centers, 2005–2010. Journal of Neuropathology & Experimental Neurology.

[ref-2] Bhasin M, Raghava GP (2004). Classification of nuclear receptors based on amino acid composition and dipeptide composition. Journal of Biological Chemistry.

[ref-3] Breiman L (2001). Random forests. Machine Learning.

[ref-4] Charoenkwan P, Ahmed S, Nantasenamat C, Quinn JMW, Moni MA, Lio P, Shoombuatong W (2022). AMYPred-FRL is a novel approach for accurate prediction of amyloid proteins by using feature representation learning. Scientific Reports.

[ref-5] Charoenkwan P, Kanthawong S, Nantasenamat C, Hasan MM, Shoombuatong W (2021). iAMY-SCM: improved prediction and analysis of amyloid proteins using a scoring card method with propensity scores of dipeptides. Genomics.

[ref-6] Chen J, Cheong HH, Siu SWI (2021). xDeep-AcPEP: deep learning method for anticancer peptide activity prediction based on convolutional neural network and multitask learning. Journal of Chemical Information and Modeling.

[ref-7] Chen T, Guestrin C (2016). XGBoost: A scalable tree boosting system.

[ref-8] Chen J, Lu Y, Yu Q, Luo X, Adeli E, Wang Y, Lu L, Yuille AL, Zhou Y (2021). TransUNet: transformers make strong encoders for medical image segmentation.

[ref-9] Chen X, Nguyen BP, Chui CK, Ong SH (2017). An automatic framework for multi-label brain tumor segmentation based on kernel sparse representation. Acta Polytechnica Hungarica.

[ref-10] Chen S, Semenov I, Zhang F, Yang Y, Geng J, Feng X, Meng Q, Lei K (2024). An effective framework for predicting drug–drug interactions based on molecular substructures and knowledge graph neural network. Computers in Biology and Medicine.

[ref-11] Chen Z, Zhao P, Li F, Leier A, Marquez-Lago TT, Wang Y, Webb GI, Smith AI, Daly RJ, Chou K-C, Song J (2018). iFeature: a Python package and web server for features extraction and selection from protein and peptide sequences. Bioinformatics.

[ref-12] Chiti F, Dobson CM (2006). Protein misfolding, functional Amyloid, and human disease. Annual Review of Biochemistry.

[ref-13] Chou K-C (2011). Some remarks on protein attribute prediction and pseudo amino acid composition. Journal of Theoretical Biology.

[ref-14] Cortes C, Vapnik V (1995). Support-vector networks. Machine Learning.

[ref-15] Dubchak I, Muchnik I, Holbrook SR, Kim SH (1995). Prediction of protein folding class using global description of amino acid sequence. Proceedings of the National Academy of Sciences of the United States of America.

[ref-16] Fix E, Hodges JL (1989). Discriminatory analysis. nonparametric discrimination: consistency properties. International Statistical Review/Revue Internationale de Statistique.

[ref-17] Hampel H, Cummings J, Blennow K, Gao P, Jack CR, Vergallo A (2021). Developing the ATX(N) classification for use across the Alzheimer disease continuum. Nature Reviews Neurology.

[ref-18] Hochreiter S, Schmidhuber J (1997). Long short-term memory. Neural Computation.

[ref-19] Ioffe S, Szegedy C (2015). Batch normalization: accelerating deep network training by reducing internal covariate shift.

[ref-20] Kingma DP, Ba J (2014). Adam: a method for stochastic optimization.

[ref-21] Li ZR, Lin HH, Han LY, Jiang L, Chen X, Chen YZ (2006). PROFEAT: a web server for computing structural and physicochemical features of proteins and peptides from amino acid sequence. Nucleic Acids Research.

[ref-22] Li Y, Ma K (2022). A hybrid model based on improved transformer and graph convolutional network for COVID-19 forecasting. International Journal of Environmental Research and Public Health.

[ref-23] López de la Paz M, Goldie K, Zurdo J, Lacroix E, Dobson CM, Hoenger A, Serrano L (2002). De novo designed peptide-based amyloid fibrils. Proceedings of the National Academy of Sciences of the United States of America.

[ref-24] Matsui Y, Tanizaki Y, Arima H, Yonemoto K, Doi Y, Ninomiya T, Sasaki K, Iida M, Iwaki T, Kanba S, Kiyohara Y (2008). Incidence and survival of dementia in a general population of Japanese elderly: the Hisayama study. Journal of Neurology, Neurosurgery & Psychiatry.

[ref-25] Maurer-Stroh S, Debulpaep M, Kuemmerer N, de la Paz ML, Martins IC, Reumers J, Morris KL, Copland A, Serpell L, Serrano L, Schymkowitz JWH, Rousseau F (2010). Exploring the sequence determinants of amyloid structure using position-specific scoring matrices. Nature Methods.

[ref-26] Mendez D, Gaulton A, Bento AP, Chambers J, De Veij M, Félix E, Magariños MP, Mosquera JF, Mutowo P, Nowotka M, Gordillo-Marañón M, Hunter F, Junco L, Mugumbate G, Rodriguez-Lopez M, Atkinson F, Bosc N, Radoux CJ, Segura-Cabrera A, Hersey A, Leach AR (2018). ChEMBL: towards direct deposition of bioassay data. Nucleic Acids Research.

[ref-27] Nguyen-Vo T-H, Teesdale-Spittle P, Harvey JE, Nguyen BP (2024). Molecular representations in bio-cheminformatics. Memetic Computing.

[ref-28] Nguyen-Vo T-H, Trinh QH, Nguyen L, Nguyen-Hoang P-U, Rahardja S, Nguyen BP (2022). iPromoter-Seqvec: identifying promoters using bidirectional long short-term memory and sequence-embedded features. BMC Genomics.

[ref-29] Nichols E, Szoeke CEI, Vollset SE, Abbasi N, Abd-Allah F, Abdela J, Aichour MTE, Akinyemi RO, Alahdab F, Asgedom SW, Awasthi A, Barker-Collo SL, Baune BT, Béjot Y, Belachew AB, Bennett DA, Biadgo B, Bijani A, Bin Sayeed MS, Brayne C, Carpenter DO, Carvalho F, Catalá-López F, Cerin E, Choi J-YJ, Dang AK, Degefa MG, Djalalinia S, Dubey M, Duken EE, Edvardsson D, Endres M, Eskandarieh S, Faro A, Farzadfar F, Fereshtehnejad S-M, Fernandes E, Filip I, Fischer F, Gebre AK, Geremew D, Ghasemi-Kasman M, Gnedovskaya EV, Gupta R, Hachinski V, Hagos TB, Hamidi S, Hankey GJ, Haro JM, Hay SI, Irvani SSN, Jha RP, Jonas JB, Kalani R, Karch A, Kasaeian A, Khader YS, Khalil IA, Khan EA, Khanna T, Khoja TAM, Khubchandani J, Kisa A, Kissimova-Skarbek K, Kivimäki M, Koyanagi A, Krohn KJ, Logroscino G, Lorkowski S, Majdan M, Malekzadeh R, März W, Massano J, Mengistu G, Meretoja A, Mohammadi M, Mohammadi-Khanaposhtani M, Mokdad AH, Mondello S, Moradi G, Nagel G, Naghavi M, Naik G, Nguyen LH, Nguyen TH, Nirayo YL, Nixon MR, Ofori-Asenso R, Ogbo FA, Olagunju AT, Owolabi MO, Panda-Jonas S, Passos VMd A, Pereira DM, Pinilla-Monsalve GD, Piradov MA, Pond CD, Poustchi H, Qorbani M, Radfar A, Reiner RC, Robinson SR, Roshandel G, Rostami A, Russ TC, Sachdev PS, Safari H, Safiri S, Sahathevan R, Salimi Y, Satpathy M, Sawhney M, Saylan M, Sepanlou SG, Shafieesabet A, Shaikh MA, Sahraian MA, Shigematsu M, Shiri R, Shiue I, Silva JP, Smith M, Sobhani S, Stein DJ, Tabarés-Seisdedos R, Tovani-Palone MR, Tran BX, Tran TT, Tsegay AT, Ullah I, Venketasubramanian N, Vlassov V, Wang Y-P, Weiss (2019). Global, regional, and national burden of Alzheimer’s disease and other dementias, 1990–2016: a systematic analysis for the global burden of disease study 2016. The Lancet Neurology.

[ref-30] Niu M, Li Y, Wang C, Han K (2018). RFAmyloid: a web server for predicting Amyloid proteins. International Journal of Molecular Sciences.

[ref-31] Orlando G, Silva A, Macedo-Ribeiro S, Raimondi D, Vranken W (2019). Accurate prediction of protein beta-aggregation with generalized statistical potentials. Bioinformatics.

[ref-32] Palato LM, Pilcher S, Oakes A, Lamba A, Torres J, Ledesma Monjaraz LI, Munoz C, Njoo E, Rinauro DJ, Menefee KA, Tun A, Jauregui BL, Shapiro S, Nossiff OH, Olivares E, Chang K, Nguyen V, Nogaj LA, Moffet DA (2019). Amyloidogenicity of naturally occurring full-length animal IAPP variants. Journal of Peptide Science.

[ref-33] Pham HN, Do TTT, Jie Chan KY, Sen G, Han AYK, Lim P, Loon Cheng TS, Nguyen QH, Nguyen BP, Chua MC (2019). Multimodal detection of Parkinson disease based on vocal and improved spiral test.

[ref-34] Prabakaran R, Rawat P, Kumar S, Michael Gromiha M (2021). ANuPP: a versatile tool to predict aggregation nucleating regions in peptides and proteins. Journal of Molecular Biology.

[ref-35] Rambaran RN, Serpell LC (2008). Amyloid fibrils: abnormal protein assembly. Prion.

[ref-36] Saravanan V, Gautham N (2015). Harnessing computational biology for exact linear B-cell epitope prediction: a novel amino acid composition-based feature descriptor. OMICS: A Journal of Integrative Biology.

[ref-37] Thangakani AM, Kumar S, Nagarajan R, Velmurugan D, Gromiha MM (2014). GAP: towards almost 100 percent prediction for β-strand-mediated aggregating peptides with distinct morphologies. Bioinformatics.

[ref-38] Thi Phan L, Woo Park H, Pitti T, Madhavan T, Jeon Y-J, Manavalan B (2022). MLACP 2.0: an updated machine learning tool for anticancer peptide prediction. Computational and Structural Biotechnology Journal.

[ref-39] Tolles J, Meurer WJ (2016). Logistic regression: relating patient characteristics to outcomes. The Journal of the American Medical Association.

[ref-40] Vrana JA, Gamez JD, Madden BJ, Theis JD, Bergen HR, Dogan A (2009). Classification of amyloidosis by laser microdissection and mass spectrometry–based proteomic analysis in clinical biopsy specimens. Blood.

[ref-41] Wang M, Cui X, Li S, Yang X, Ma A, Zhang Y, Yu B (2020). DeepMal: accurate prediction of protein malonylation sites by deep neural networks. Chemometrics and Intelligent Laboratory Systems.

[ref-42] Wang H, Shang Y, Wang E, Xu X, Zhang Q, Qian C, Yang Z, Wu S, Zhang T (2022). MST1 mediates neuronal loss and cognitive deficits: A novel therapeutic target for Alzheimer’s disease. Progress in Neurobiology.

[ref-43] Wang H, Yan Z, Yang W, Liu R, Fan G, Gu Z, Tang Z (2025). A strategy of monitoring acetylcholinesterase and screening of natural inhibitors from Uncaria for Alzheimer’s disease therapy based on near-infrared fluorescence probe. Sensors and Actuators B: Chemical.

[ref-44] Wei L, Zhou C, Chen H, Song J, Su R (2018). ACPred-FL: a sequence-based predictor using effective feature representation to improve the prediction of anti-cancer peptides. Bioinformatics.

[ref-45] Wu T, Li P, Sun J, Nguyen BP (2024). Adaptive edge prior-based deep attention residual network for low-dose CT image denoising. Biomedical Signal Processing and Control.

[ref-46] Yang R, Liu J, Zhang L (2023). ECAmyloid: an amyloid predictor based on ensemble learning and comprehensive sequence-derived features. Computational Biology and Chemistry.

[ref-47] Zhao J, Jiang H, Zou G, Lin Q, Wang Q, Liu J, Ma L (2022). CNNArginineMe: a CNN structure for training models for predicting arginine methylation sites based on the one-hot encoding of peptide sequence. Frontiers in Genetics.

